# Dissociation between the Activity of the Right Middle Frontal Gyrus and the Middle Temporal Gyrus in Processing Semantic Priming

**DOI:** 10.1371/journal.pone.0022368

**Published:** 2011-08-04

**Authors:** Ilan Laufer, Michiro Negishi, Cheryl M. Lacadie, Xenophon Papademetris, R. Todd Constable

**Affiliations:** 1 Department of Diagnostic Radiology, Yale University, New Haven, Connecticut, United States of America; 2 Department of Biomedical Engineering, Yale University, New Haven, Connecticut, United States of America; University of Zaragoza, Spain

## Abstract

The aim of this event-related functional magnetic resonance imaging (fMRI) study was to test whether the right middle frontal gyrus (MFG) and middle temporal gyrus (MTG) would show differential sensitivity to the effect of prime-target association strength on repetition priming. In the experimental condition (RP), the target occurred after repetitive presentation of the prime within an oddball design. In the control condition (CTR), the target followed a single presentation of the prime with equal probability of the target as in RP. To manipulate semantic overlap between the prime and the target both conditions (RP and CTR) employed either the onomatopoeia “oink” as the prime and the referent “pig” as the target (OP) or vice-versa (PO) since semantic overlap was previously shown to be greater in OP. The results showed that the left MTG was sensitive to release of adaptation while both the right MTG and MFG were sensitive to sequence regularity extraction and its verification. However, dissociated activity between OP and PO was revealed in RP only in the right MFG. Specifically, target “pig” (OP) and the physically equivalent target in CTR elicited comparable deactivations whereas target “oink” (PO) elicited less inhibited response in RP than in CTR. This interaction in the right MFG was explained by integrating these effects into a competition model between perceptual and conceptual effects in priming processing.

## Introduction

Semantic priming is the facilitation of the response to a word (target) which is preceded by a semantically or conceptually related word (prime) and entails response suppression attributed to the priming effect which results in faster processing of primed stimuli. For more information on priming see [1], [2]. One of the explanations for this neural suppression is that the recognition threshold for the primed targets is lowered by the spreading of activation from the prime [1], [3], [4], [5].

Modulation of the hemodynamic response to primed relative to unprimed targets was found in a variety of areas encompassing temporo-parietal regions, inferior prefrontal cortices as well as bilateral middle frontal gyri and anterior cingulate [3], [4], [6]–[15]. In this study we focused on two regions typically found to be engaged in priming processing, the middle temporal gyrus (MTG) [8], [11], [13], [16]–[18] and the middle frontal gyrus (MFG) [2], [8], [11], [12], [18], [19].

The MTG (Brodmann areas [BAs] 21/22, 37, 39) has been associated with increased activation for related words in both the left and right hemispheres [8], [11], [13] but also with increases for unrelated vs. related words [17]. The left mid-posterior MTG has been thought to serve as storage of lexical representations, and was found to be engaged in tasks involving semantic judgments and categorization. Activity in this region is increased as a function of intelligibility and the number of words processed per trial [20]. Recently, activity in the posterior left MTG has been linked to executive semantic control since it was associated with greater activations in conditions that required increased retrieval effort and demanding semantic decisions in the context of priming [21], [22].

In the MFG (BAs 46/9) increases have been observed bilaterally in response to unrelated vs. related word pairs [8], [12], [19]. Decreased activation in the right MFG (BA 8) has been linked to a right hemisphere advantage in processing categorical vs. associative relations because of the more efficient concept retrieval of perceptually similar objects [2]. This stands in contrast to the increased activation in the right MFG (BAs 46/9) reflecting an enhanced effort in processing categories vs. associations [22] and to enhanced activity in this region elicited by increased semantic overlap between abstract words [11]. The diverse activation pattern in the right MFG has been thought to reflect executive semantic processes and the relative ease of retrieval or search of the semantic network [2], [8], [12], [19], [23], [24]. Thus, regardless of the direction of change in activation, previous research indicates that activation in the right MFG is modulated by categorical relations.

In the current event-related functional magnetic resonance imaging (fMRI) study we examined whether repetition suppression [25]–[33] in brain activity for targets that were primed by multiple primes was sensitive to prime-target conceptual relations. The aim was to test whether the right MFG and the left MTG would show differential sensitivity to the effect of the magnitude of semantic overlap between the prime and target on repetition priming. These regions were chosen because they are both involved in priming and executive semantic control, while the left MTG has also been found to be implicated in the release of adaptation effects [34]–[36] typically caused by introducing a physically novel stimulus following repetitive stimulation [29], [37], [38]. Therefore, while the right MFG has been found to be sensitive to conceptual priming effects, the left MTG has been shown to be sensitive to both perceptual and conceptual effects.

Perceptual priming alludes to the physical features of the stimulus while conceptual priming is linked to the semantic or abstract features of the stimulus. For a distinction between perceptual and conceptual priming see [39], [40]. Thus, while the right MFG was expected to be sensitive to the magnitude of the semantic overlap between the target and the prime, in the left MTG it was expected that release of adaptation caused by perceptual effects would mask any conceptual effects modulated by the degree of semantic overlap between the target and the prime.

Semantic overlap between the prime and the target was manipulated in this study by changing the order of the prime and the target [41]. In brief, in that study [41] it was shown that sound-word pairings (e.g. oink-pig) elicited a more robust priming effect than word-sound pairings. This is because when “oink” precedes its referent “pig” the semantic overlap between them increases more than in the reversed case. Accordingly, in the current study, in both RP and CTR the greater semantic overlap was achieved by oink-pig (OP) and the smaller overlap by pig-oink (PO). All the stimuli used in the present study were naturally produced words. Repetition priming (RP) was induced by using a conventional oddball design in which the target appeared randomly after several repeated presentations of the prime. In the control condition (CTR) targets followed a single presentation of the prime.

In view of the above we have hypothesized that in the experimental condition (RP) a more robust priming in case of OP vs. PO would be evident in the right MFG but not in the left MTG. Specifically, a Condition (RP, CTR)×Stimulus (“oink”, “pig”) interaction was expected in the right MFG but not in the left MTG for the following reasons. In the right MFG “pig” targets (OP) would elicit continuous conceptual adaptation in RP but would also elicit decreased activation in CTR because of the presentation of the preceding related prime. In contrast, “oink” targets would elicit conceptual release of adaptation leading to increased activation in RP and decreased activation in CTR (since the target was primed).

Previously, a widely distributed network including temporal, premotor and prefrontal regions was found to be involved in processing both perceptual and conceptual features of repeated auditory stimuli [39]. Thus, the novelty of the current study is in demonstrating dissociated activity between two regions that are both involved in conceptual retrieval of information but are differentially affected by perceptual effects. The results to follow will demonstrate that the influence of the extent of semantic overlap on repetition priming was found for the right MFG but not for the MTG bilaterally.

## Methods

### Ethics Statement

This study has been approved by Yale University Human Investigation Committee (HIC). Informed written consent was obtained from all participants in accordance with the guidelines of the Yale Human Research Protection Program (HRPP).

### Subjects

Twenty one right-handed adult healthy subjects, native speakers of English, 6 women, participated in the study. Subjects ranged in age from 19 to 39 (mean 25.4±6.2 (± standard deviation [SD])).

### Experimental protocol

#### Procedure and task

Spoken words were presented in an fMRI event-related design while participants performed a silent counting task. The subjects were instructed to silently count every stimulus, press a button when they reached 100 and then restart counting from one again while they listened to the stimuli through headphones presented in 95 dB SPL. The counting task was chosen for two reasons. The first is related to the fact that the target in our study was embedded within oddball sequences and was a rare event in both RP and CTR (see “Procedure and task” in Methods). Thus, the purpose of the counting task was to diminish the effect of deviant processing especially on right prefrontal cortex since this region is implicated in both attentional capture and contrast enhancement [42], [43]. The second reason was to ensure the vigilance of the subject during the presentation of the stimuli and alleviate boredom effects in each of the runs. Therefore, the counting task was emphasized by asking subjects to report the number they reached at the end of each run.

**Figure 1:**
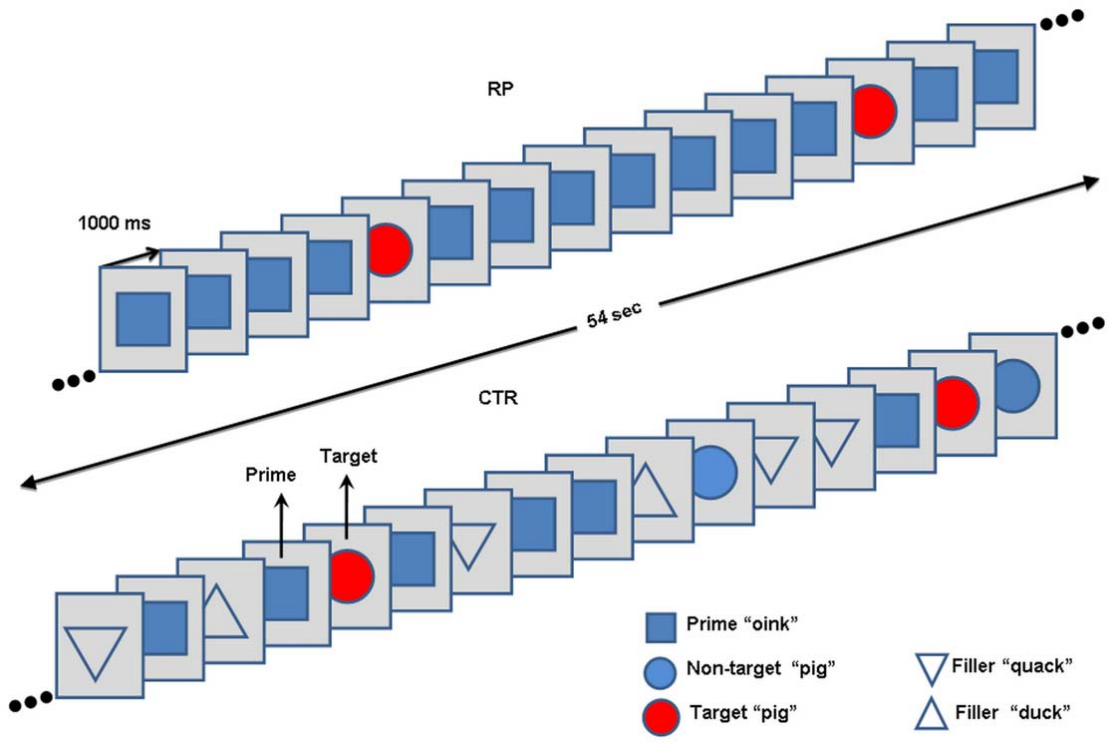
Experimental design: schematic diagram of sequences used in RP (upper panel) and CTR (lower panel). The example here pertains to the case when “pig” served as the target (onomatopoeia “oink” primes pig: OP), but an identical scheme of stimulation (not shown) was used when “oink” served as the target and “pig” (PO) as either the repetitive prime (RP) or the single prime preceding the target (CTR). The stimuli “duck” and “quack” served as “filler” stimuli and were used to prevent immediate proximity between “oink” and “pig” within the varying segments in CTR so that the target would have the same probability of occurrence as in RP (∼17%). A complete run was divided into eight 54 msec segments separated by a 2 sec interval, each containing 54 stimuli (targets, primes and in CTR also fillers) with an SOA of 1 sec. Stimulus duration was 330 msec. For each condition (RP, CTR) there were two different sequences, one for each target-type. The four sequences were first ordered according to a Latin-square design to minimize carry-over effects and then mirror-imaged for the second part of the run. Each run started and ended with a period of 30 sec duration of white noise that served as the baseline. Note that the study used auditory stimuli (not visual), i.e., naturally produced speech stimuli (the words: “oink”, “pig”, “duck” and “quack”) and that there were three exemplars of each word.

Participants completed six runs, each comprised a total of 432 stimuli occurring at an SOA of 1 sec and lasted approximately 9 min. Each run included both experimental and control conditions, each of which included both PO (target: “oink”) and OP (target: “pig”) sequences (Fig. 1). It is noteworthy that only two stimuli were selected as prime and target (“oink” and “pig”) throughout the entire session while in priming studies several different prime-target pairs are usually employed. However, we wanted to control for physical stimulus properties so that the target in RP would be compared with its physical equivalent target in CTR. Therefore, we reversed between the roles of primes and targets as commonly done in Mismatch negativity (MMN) “identity” studies [44]. In these studies, as in ours, the aim is to minimize physical differences between the relevant contrasted stimuli (in identity studies, the deviant stimulus and the standard stimulus). The reversal between the roles of prime and target in our study resulted in repeating each condition (RP and CTR) twice, once when “oink” was the prime and “pig” the target and vice-versa in a second sequence. Therefore, inclusion of additional prime-target pairs would have been impractical since it would have substantially prolonged the overall duration of the study.

Figure 1 displays the stimulus sequences corresponding to OP where “pig” was the target. The experimental condition (Fig. 1, upper panel) was designed as a classic oddball sequence in which either “pig” or “oink” as targets were embedded within a homogenous train of a repeated prime (either “oink” or “pig”, respectively). The probability of occurrence of the target was approximately 17%. We hereafter refer to the experimental condition as RP (which stands for “repeated prime”). In the control condition (CTR) (Fig. 1, lower panel) prime-target pairs (“oink”-“pig”, respectively, or vice versa) were embedded within a sequence such that “pig”, “oink”, “duck” and “quack” appeared in a quasi-random order. Thus the stimuli “duck” and “quack” served in CTR as “filler” stimuli so that “oink” and “pig” would only occasionally occur adjacent to each other as a prime-target pair. However, “duck” and “quack” never appeared immediately adjacent to each other. The probability of occurrence of the target immediately preceded by the prime in CTR was equal to that of its counterpart in RP (approximately 17%) (Fig. 1).

The first half of each session consisted of three runs that were different from each other. The second half of the session consisted of three runs that were identical to those in the first half, except that the order of the runs was the mirror image of that in the first half. Each run comprised four sequence types (condition [RP, CTR]×target [“pig”, “oink”]) each with 54 stimuli appearing quasi-randomly. The order of the four sequences was permutated in the first half of each run using the Latin square method (e.g. 2, 1, 4, 3). In the second half of the run the order of the four sequences was mirror-imaged (i.e. 3, 4, 1, 2) but different sequences were used. Thus, there were in total eight sequences in a run since for each condition there were two different versions (e.g. 2 different versions of the RP sequence with target “pig” in the same run). The two versions of each sequence differed only in the order of stimuli presentation within the sequence. In total, there were 12 different versions of RP and CTR sequences (2 versions×2 targets×3 runs). Each run started and ended with a 30 sec. continuous interval of white noise. There were 2 sec. silence intervals between sequences as well as between the white noise and the initial and ending sequence of each run. The target (either “pig” or “oink”) was primed 9 times out of a total of 12–15 of its appearances in the sequence (see Table 1 for the probability of occurrence of each stimulus type comprised in CTR sequences).

**Table 1:** Probabilities of the stimuli in the CTR condition.

Stimulus Type	Corresponding geometrical shapes and colors	Probability
Fillers	Triangles	47%
Targets	Red circles	17%
Primes immediately preceding targets	Blue squares preceding red circles	17%
Non-targets (targets occurring without preceding primes)	Blue circles	10%
Isolated primes	Blue squares between fillers	6%
Primes following themselves	Blue squares	3%

Probabilities are given relative to a sequence containing 54 stimuli and represent averages collapsed across all 12 different versions of the CTR sequence. Geometrical shapes and colors as in Fig. 1.

#### Stimuli

Multiple repetitions of each of the words “pig”, “oink”, “quack” and “duck” produced by a male native speaker of English were recorded. Three exemplars for each word (e.g. oink1, oink2, oink3) were selected (out of a pool of 24 recordings per stimulus) on the basis of acoustic similarity. The parameters that were used to select similar exemplars for each word included the shape of the spectrogram at the voice onset, vowel durations, pitch and formant values (Hz) of the first three formants (Table 2). The stimuli (“pig”; “oink”; “quack”; “duck”) were truncated to 330 msec and normalized to the same loudness level by using Adobe Audition 2.0 trial version software package. Spectral analysis of the stimuli was conducted by PRAAT software (http://www.fon.hum.uva.nl/praat/).

**Table 2:** Pitch and mean formant frequencies of the main speech stimuli (in Hz).

Stimulus	F_0_	F_1_	F_2_	F_3_
pig1	102	453	2090	2578
pig2	102	483	2115	2644
pig3	104	502	2086	2631
oink1	108	541	1530	2712
oink2	106	517	1753	2755
oink3	103	543	1554	2739

F_0_ = pitch. F_1_, F_2_ and F_3_ indicate the mean frequencies (Hz) of the first, second and third formants, respectively, across the length of the initial CV or diphthong.

The reason for using three tokens for each stimulus was to control as much as possible for acoustic factors which could confound the semantic relationship between the prime and the target. Specifically, using three different exemplars for each stimulus diminished the likelihood of a contingency developing within a specific prime-target pair because of an uncontrolled acoustic facet associated with either the target or the prime. Stimulus presentation was carried out by E-Prime (Psychology Software Tools, Inc., Pittsburgh, PA; http://www.pstnet.com/prime).

#### Evaluation of priming effects

In a post-test out of the scanner session the same group of subjects that participated in the fMRI scans listened to the same four sequence types (condition [RP, CTR]×target [“pig”, “oink”]) delivered in the scanner. However, the behavioral sequences were shorter relative to those used during the scan and contained 8 instead of 9 presentations of the target (p = .16). In the behavioral session data were gathered from 20 subjects. Subjects were asked to detect any transition from “oink” to “pig” or vice versa and respond by a button press. We did not acquire behavioral responses during scanning (e.g. [45]) to diminish the involvement of two confounds that could potentially affect priming processing. The first was motor-related brain activity associated with button-pressing. The second was deviance-related processing. Specifically, since the target was a rare event within a sequence, overt target detection could have enhanced the effect of attentional processes on prefrontal and temporal activation [46] relative to the counting task which did not require active discrimination between the target and any of the preceding stimuli [31].

### fMRI Scanning Technique and Data Analysis

#### Data Acquisition Parameters

Data were collected on a 3T Siemens Trio MRI scanner. Each study began with two localizers: a 3-plane localizer and a multiple-slice sagittal localizer. These were followed by the acquisition of twenty five 6 mm T1-weighted axial slices (TR = 300 msec, TE = 2.47 msec, flip angle = 60 degrees, FOV = 220 mm, 256×256 acquisition matrix). For each subject, 6 functional imaging runs were collected with slices in the same locations as the anatomical T1-weighted data. Functional images were recorded using a gradient-echo EPI sequence (TR = 1550 msec, TE = 30 msec, flip angle = 80 degrees, FOV = 220 mm, 64×64 acquisition matrix). Each functional run involved the acquisition of 347 volumes. Images were converted to analyze format and the first six volumes of each functional series were removed to account for the approach to steady-state magnetization, leaving 341 volumes for analysis.

#### Preprocessing and Image Analysis

Using sinc interpolation, the data from each slice were adjusted for slice acquisition time and then motion corrected using SPM5 (http://www.fil.ion.ucl.ac.uk) for 6 rigid body motions (displacement in the x, y, z direction and rotation: for pitch, roll, yaw). Flags were set for masking such that a pixel was set to zero for every time point if it moved outside the volume in any time point. Functional image data were motion corrected by realigning all volumes to the first volume in the middle run.

Individual subject data (responses to the targets, primes and “filler” stimuli) were analyzed using a Generalized Linear Model on each voxel in the entire brain volume. The data were normalized to a signal measure of 100 and spatially smoothed with an 8 mm Gaussian kernel to account for variations in the location of activation across participants. The output maps were normalized beta-maps in the acquired space (3.44 mm×3.44 mm×6 mm).

To take these data into a common reference space, three registrations were calculated using the Yale BioImage Suite software package (http://www.bioimagesuite.org) following the same procedures as described in [29].

### Data analyses

#### Within subject analyses

The following types of maps from an event-related analysis were calculated separately for the “oink” and “pig” stimuli: (1) *RP target maps* computed from RP sequences extracting the response to targets (red circles, Fig. 1, upper panel) (2) *Repeated prime maps* computed from RP sequences extracting the response to the repeated primes (blue squares, Fig. 1, upper panel) (3) *CTR target maps* computed from CTR sequences extracting the response to targets (red circles, Fig. 1, lower panel) (4) *CTR prime maps* computed from CTR sequences extracting the responses to primes and physically equivalent unprimed stimuli in the counterpart sequence. For example, when “pig” was the target in OP, all “oink” primes (blue squares, Fig. 1, lower panel) in OP and all “oink” unprimed targets (non-targets) in PO (equivalent to the blue circles in Fig. 1, lower panel) were assigned to the same regressor. The “duck” and “quack” filler stimuli (triangles, Fig. 1, lower panel) were entered into a separate regressor. We examined the blood oxygenation level-dependent (BOLD) signal changes for each stimulus type compared to baseline of white noise inserted at the beginning and end of each run for a period of 30 sec.

### Across subject analysis

#### ROI selection

ROIs were chosen in the MTG and MFG bilaterally based on a functional contrast map between CTR targets and CTR primes. The purpose of using this contrast was to extract brain regions implicated in priming processing without the confounding effects of release of adaptation [47] elicited by RP targets, and of the Stimulus factor. The latter could have biased the selection of ROIs according to dominant physical features of either “pig” or “oink”. Therefore, the contrast CTR targets vs. CTR primes was calculated across both “pig” and “oink” stimuli and masked with a Condition (RP, CTR)×Event-type (target, prime) interaction to confine the resultant regions to the boundaries dictated by the interaction alone. To assess the effect of the magnitude of semantic overlap between the target and the prime on repetition priming, RP targets were contrasted with CTR targets in each of the selected ROIs. Both contrasts, CTR targets vs. CTR primes (priming effect map) and RP targets vs. CTR targets (ROI analyses) are second-level contrasts derived from the Condition×Event-type interaction that was used as a mask.

**Figure 2:**
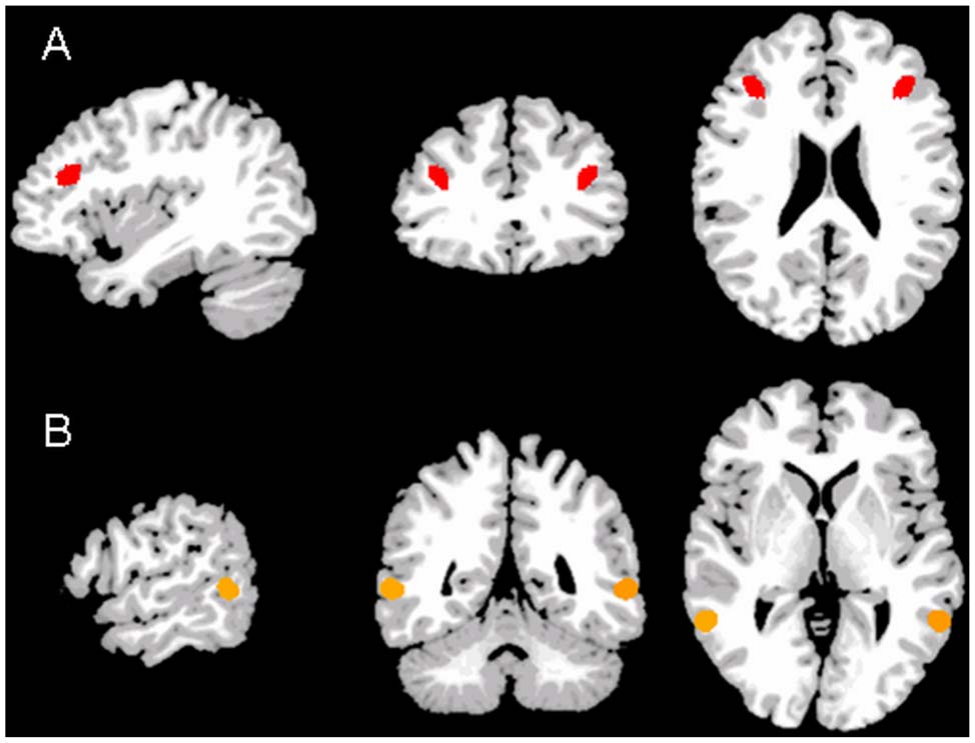
The MFG (A) and MTG (B) ROIs. The figure presents the selected ROIs in three orthogonal views (sagittal, coronal, axial). ROIs were extracted from a contrast map between CTR targets vs. CTR primes. The x,y,z Talairach coordinates for the right MFG and MTG are set on the center of mass coordinates of each ROI as follows: x = 36, y = 30, z = 21; x = 59, y = −50, z = 5, respectively. The left ROIs were achieved by flipping the right ones. The functional ROIs were superimposed on a reference anatomical image (Holmes et al., 1998). Display follows radiological convention.

For the mask a voxel-wise threshold of *p*<0.05 (*F*(1,20) = 4.35) and a cluster threshold of *p*<0.05 was used to correct for multiple comparisons. To further minimize stimulus effects, the Event-type (target, prime)×Stimulus (“oink”, “pig”) interaction map was subtracted from the mask (the Stimulus main effect as well as the Condition×Stimulus interaction were not significant at *p*<0.05). The resultant masked contrast map (CTR target vs. CTR prime) was thresholded at *p*<0.001 (uncorrected). This functional contrast map showed negative BOLD responses in the right hemisphere. The relevant regions to the hypothesis of the study, namely the MFG (BAs 46/9) and MTG (BAs 21/37) were chosen from this contrast map and are shown in Fig. 2. The left MFG and MTG were not significant at the threshold used and were achieved by flipping the ROIs from the right hemisphere (Fig. 2).

The coordinates of the center of mass for the right MFG were: x = 36, y = 30, z = 21 and the volume of this region was 920 mm^3^. The coordinates of the center of mass for the right MTG were: x = 59, y = −50, z = 5 and the volume of this region was 792 mm^3^. Coordinates are based on a conversion [48] from Montreal Neurological Institute (MNI) space to Talairach coordinates [49] Beta values from single-subject data were extracted from these ROIs and were then subjected to a two-way repeated-measures analysis of variance (ANOVA) using SPSS 16.0 software (SPSS, Chicago, IL) with Condition (RP, CTR) and Stimulus (“oink”, “pig”) as factors. Separate analyses were conducted for the MTG and MFG bilaterally. Thus, overall, four different ANOVAs were run. Only the percent signal change values for targets were included in these ANOVAs. Greenhouse-Geisser corrections were applied to account for non-sphericity.

## Results

### Behavioral results

#### Detection rates and reaction times

Detection rates were approximately 95% for each experimental condition. A 2 by 2 within-subjects repeated measures ANOVA with factors Condition (RP, CTR) and Target-type (“pig”, “oink”) was performed on the reaction times (mean [msec] ± standard error for RP “pig” = 485±16; RP “oink” = 440±18; CTR “pig” = 420±19; CTR “oink” = 407±19). A main effect of Condition (RP>CTR, *F*(1,19) = 20.594, *p*<0.001) and Target-type (“pig”>“oink”, *F*(1,19) = 9.779, *p*<0.01) were found, but there was no significant interaction between these factors.

#### Silent counting

The subjects were instructed to count every stimulus, press a button when they reached 100 and then restart counting from one again. The subjects were also asked to report the number they have reached at the end of their count. Since there were 432 stimuli per run there were 4 full cycles of counting. The average number (± SD) of trials reached at button press across runs and across subjects as a function of the counting cycle (1 through 4) was as follows: 100.6 (±6.9); 201.6 (±17.0); 300.3 (±16.3); and 399.6 (±17.8), respectively. The average of the number the subjects reported at the end of their count, averaged across subjects and runs, was 35.4 (±17.8). These data indicate that the subjects were indeed counting to 100 during each run.

### ROI analyses

**Figure 3:**
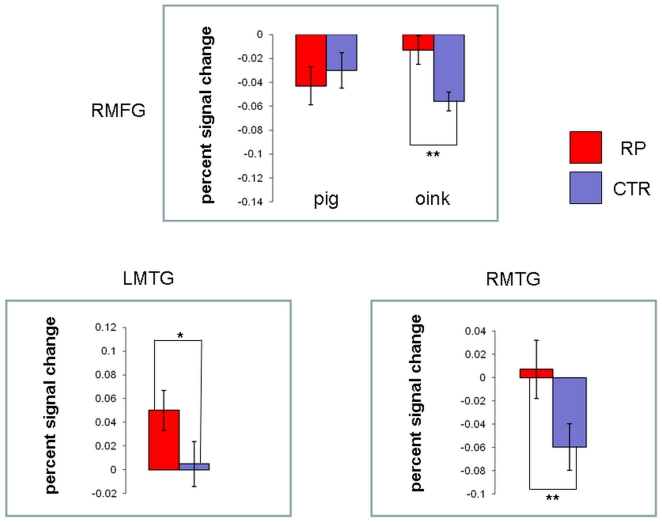
Responses to targets in RP and CTR within each of the ROIs. Upper panel: the Condition (RP, CTR)×Stimulus (oink”, “pig”) interaction in the right MFG. Note the different responses to “oink” in RP relative to CTR and the more similar activation levels for “pig” primes in these conditions. Lower panels: the condition effect (RP>CTR) in the left and right MTG (left and right panels, respectively). Note the deactivation in CTR in the right MTG and the positive increased activation in RP relative to CTR in the left MTG. R = right; L = left. Error bars depict the standard error. *p<0.05; **p<0.01.

To assess the effect of semantic overlap (i.e. OP vs. PO) on repetition priming, RP targets were compared to CTR targets for each of the target stimuli (“oink”, “pig”) and ROIs. It is noteworthy that the paired t-test comparisons were performed between physically identical stimuli. This was possible because of the reversal between the roles of the prime and target in each of the conditions (RP and CTR). The percent signal change in all ROI analyses displayed in Fig. 3 was derived from the signal corresponding to targets as compared to baseline.

In the right and left MTG, respectively, only a significant main effect of condition (RP>CTR, *F*(1,20) = 9.629, *p*<0.01; right; RP>CTR, *F*(1,20) = 5.174 *p*<0.05, left) was found. In the right MFG a significant Condition (RP, CTR)×Stimulus (“oink”, “pig”) interaction (*F*(1,20) = 8.373, *p*<0.01) was revealed (Fig. 3). Paired t-test comparisons indicated a significantly elevated response to RP “oink” targets relative to CTR “oink” targets, (*t*(20) = 3.229, *p*<0.01). However, in the left MFG no significant effects were found (*p*>0.05). Note that in Fig. 3 percent signal change values were derived from a signal corresponding to target and primes as compared to baseline, respectively.

## Discussion

### fMRI results

The aim of the current study was to test whether the right MFG (BAs 46/9) and the left MTG (BAs 21/37) would show differential sensitivity to the effect of conceptual relations on repetition priming. The magnitude of semantic overlap between the target and the prime was manipulated by reversing primes and targets (i.e.,wide overlap: oink – pig [OP]; narrow overlap: pig – oink [PO]). Repetition priming was induced via a conventional oddball design, in which the target appeared randomly after several repeated presentations of the prime (RP). In the control condition (CTR), targets followed a single presentation of the prime. Parameter estimates were extracted for targets and primes in the RP and CTR condition from four regions of interest (i.e., left and right MFG and MTG) and analysed using repeated-measures analyses of variance (ANOVA).

The hypothesis of our study was that in the experimental condition (RP) a more robust conceptual priming effect would be evident in the right MFG but not in the left MTG for OP vs. PO. The rationale behind this hypothesis was that semantic overlap in OP is greater than in PO [41] and the right MFG is sensitive to categorical vs. associative priming probably because of a greater perceptual similarity between categorical pairs [2], [22]. The hypothesis of our study was confirmed. Only in the right MFG, a Condition (RP, CTR)×Stimulus (“oink”, “pig”) interaction was revealed. The interaction was due to similar levels of deactivation elicited by “pig” targets (OP) whereas “oink” targets (PO) elicited differential levels of deactivation between RP and CTR (Fig. 3).

As hypothesized, in the left and right MTG no difference was found between OP and PO. In both regions only a main effect of condition was revealed (RP>CTR). However, in the right MTG activation levels in RP and CTR became more negative relative to the corresponding activations in the left MTG (Fig. 3). This could be explained by release of adaptation effects [29], [37], [38] that were more prominent in the left MTG [34]–[36] than in the right MTG caused by the introduction of the target. Nevertheless, both in the right and left MTG targets were associated with enhanced activations in RP regardless of target identity (Fig. 3).

However, release of adaptation could not account for the deactivation observed in the right MTG in CTR as well as in the right MFG both in RP and CTR (Fig. 3). To explain these deactivations it is important to note that the ROIs were selected from a contrast map within CTR (targets vs. primes) to extract the priming effect un-confounded by release of adaptation effects. This contrast map revealed negative BOLD responses in the ROIs on the right side and therefore the deactivations observed in the right MFG and MTG in response to CTR targets (Fig. 3) were to be expected. We suggest that the same process affected deactivations observed in CTR both in the right MFG and MTG, namely, extraction of event regularities [50], [51].

#### Deactivations in the right MFG and MTG

It was previously found that the right MFG is engaged in the verification of sequence regularity [50], [51]. This region was associated with decreases when predictions were confirmed [51] whereas violations of expectations were shown to be associated with increases in activation [50], [51]. Increased activation in the dorsolateral prefrontal cortex (DLPFC, BAs 46/9) may reflect restructuring of a forward model when sequential violations are detected [50].

Accordingly, in our study, when the target was introduced in CTR it validated the expectation elicited by the preceding prime, e.g., “pig will be followed by oink”. Thus, concurrent with target introduction the prediction was verified, reconfiguration costs were minimal and deactivation in the DLPFC ensued. Similarly, it was previously shown that repetition suppression reflects the reduction of prediction error when an event is expected [32]. Hence, the deactivations observed in the right MFG in the CTR condition may reflect the effect of a verification process that masked the semantic priming effect or alternatively, both effects were either additive or interactive while eliciting the observed deactivation (Fig. 3).

The deactivation observed in the right MTG in the CTR condition (Fig. 3) could be explained in a similar manner. The right MTG was previously found to be implicated in extraction of sequence regularity [50], [52] but not in processing erroneous expectations [50]. However, as in the right MFG, deactivation in the right MTG was also observed but only in CTR and not in RP (Fig. 3). This deactivation may imply that this region was also sensitive to confirmed expectations as with the right MFG.

#### Dissociated activity between MFG and MTG

The primary focus of this study was to show dissociation between the right MFG and left MTG in processing the effect of the magnitude of semantic overlap on repetition priming (RP). While dissociated activity was found in the right MFG between OP and PO it was not found in the MTG (Fig. 3). Specifically, in the right MFG target “pig” (OP) in both RP and CTR elicited comparable levels of deactivations whereas different deactivations levels were found for target “oink” (PO) in RP and CTR (Fig. 3). This is because in PO semantic overlap was smaller than in OP causing target “oink” to be less expected than target “pig”. Therefore, following multiple repetitions of the prime, target “oink” in RP elicited less deactivation relative to CTR reflecting release of conceptual adaptation (Fig. 3).

Thus, it is the RP condition that distinguished between OP and PO in the right MFG. This means that multiple repetitions of the prime (RP) were required to establish either continuation of conceptual adaptation (target “pig”) or conceptual release of adaptation (target “oink”) (Fig. 3). Hence, the deactivations in the right MFG observed in RP were modulated by conceptual effects and could not be explained by the fact that the ROIs were selected from a negative BOLD map.

Contrary to previous findings [50] the present study indicates that both the right MFG and MTG may be engaged in sequence verification but whereas the right MFG was associated with increased depth of processing of conceptual relations between stimuli (while deactivations reflected the magnitudes of those relations) the right MTG was insensitive to these effects.

#### Conceptual adaptation effects in the right MFG

The results found in the right MFG described above could be accounted for by an adaptation model that also accounts for semantic stimulation and conceptual adaptation [53]. According to this model semantic information activates stimulus selective cells in cortical sensory areas as well as in areas that perform semantic processing. Repetitive stimuli are mapped onto suppressed parts of the relevant cortical maps leading to habituation effects. Novel stimuli which represent a large change are activating regions outside the suppressed map leading to dishabituation [27], [36], [38]. According to this adaptation model the reduced suppression in the right MFG observed for “oink” in RP is the result of conceptual release of adaptation elicited by “oink” preceded by multiple “pig” primes (Fig. 3) and may indicate that the right MFG represents one of the cortical regions sensitive to conceptual adaptation effects.

#### A hierarchical model of processing priming effects

We tentatively suggest that the above findings involving the right MFG and left MTG could be congruent with a competition model between sensory and conceptual effects. Specifically, according to this model, information from the temporal lobe (left MTG) was projected to the right MFG that in turn exerted a modulatory effect on the received input. When the semantic overlap was wide (OP) a high competition ensued between sensory (local adaptation effects in the left MTG) and semantic information. In this situation, semantic information processing was enhanced in higher-level prefrontal areas (right MFG) while the information received from lower-level temporal information, i.e. release of perceptual adaptation effects, was irrelevant. This resulted in a continuing conceptual adaptation effect in the right MFG elicited by target “pig” (Fig. 3). However, when the semantic overlap was small (PO) stimulus specific adaptation effects were more prominent than conceptual effects and release of perceptual adaptation in temporal areas was relayed onto higher-level areas (right MFG) in the form of increased activation that was not suppressed by the right MFG (Fig. 3).

The feedforward process described in our model is in line with predictive coding accounts [26], [54] according to which an error signal (reflecting prediction error) is projected to a higher-level region where prediction update is generated. However, the component added in our model is that the modulatory effect exerted by the right MFG on the received error-signal is weighted according to the dominance of perceptual vs. conceptual effects elicited by the incoming stimulus.

Our model is also partly in line with the previously proposed hierarchical organization of the auditory cortex [55] according to which sensory areas are sensitive to acoustic factors whereas higher-level areas in the processing chain are insensitive to these factors. Specifically, the authors [55] summarize a hierarchical model based on previous findings [56] from a study that was confined to the temporal lobe, and suggest an expanded model. The confined model portrays a posterior-anterior gradient in acoustic insensitivity moving away from primary auditory cortex. However, the expanded model also includes prefrontal, premotor/motor, and posterior inferotemporal regions as part of multiple parallel processing pathways that radiate outward from primary auditory areas [55]. Whereas previous models underlie a trade-off between acoustic sensitivity and intelligibility, our data highlights a competition between perceptual and conceptual priming [39], [40] along the processing pipeline.

The proposed competition model is also compatible with previous findings suggesting that there is increased cross-cortical synchrony between prefrontal and temporal cortices during repeated object classification and that local adaptation models are not sufficient to account for priming effects [57]. However, the latter study demonstrated that repetition induced response changes occur earlier in prefrontal than in temporal regions and it was therefore suggested that selection and control processes in the prefrontal cortex influence object processing in the temporal cortex (see also [55]). The direction of the causal relationships between the prefrontal and temporal cortices in the context of repeated auditory classification and its dynamic changes over time is an issue for further investigation, preferentially by using effective connectivity analysis (e.g. dynamic causal modeling [58]) in the context of both meaningful stimuli and repetition priming.

### The behavioral results

The longer reaction times (RTs) in RP than in CTR could be explained by the possibility that the introduction of the RP target was associated with an unexpected change relative to the preceding repetitive sequence leading to a prolonged RT [51] in RP relative to CTR. However, contrary to expectations reaction times for target “pig” were longer than for “oink”, especially in RP. This result could be explained by contextual effects as follows.

It was recently found [51] that RT costs were not only related to the amount of change in stimuli but also to the stimulation context in which a trial appeared. Specifically, in that study RTs were more prolonged for invalid and deviant trials when preceded by more valid standard trials [51]. Context effects may have also influenced RTs in our study. Specifically, in the RP condition target “pig” (OP) violated the sequence because it was physically different than the preceding multiple primes. At the same time, however, target “pig” was also conceptually associated with the preceding primes as evident by the response deactivation in the right MFG reflecting continuous conceptual adaptation (Fig. 3). Thus, this conflict between physical deviance and conceptual relatedness in RP for “pig” may have caused the prolongation in RT for this stimulus.

Contrary to expectations, suppression of the BOLD response to target “pig” in RP was associated with the prolongation of RT to this stimulus. In both the behavioral task (outside the scanner) and the counting task (inside the scanner) the same sequences were used with an SOA of 1000 msec. Therefore, controlled processes [59] would have been expected to occur both inside and outside the scanner but to different degrees (more prominent in the latter). Thus initially it was expected that the behavioral measures would be positively correlated with the imaging data (i.e. decreased activation would be associated with reduced RTs). We suggest that the dissociation between the behavioral and imaging data would still exist if RTs were also measured during scanning since the behavioral measures and the BOLD signal in the right MFG were affected by different processes.

Specifically, in the SOA employed in the current study (SOA = 1000 msec) controlled processes, such as semantic matching and expectancy generation [4], [16], [59], [60] which take time to develop, affected behavioral measures such as reaction times (RTs) [17]. In contrast, conceptual adaptation as described in [53] (see “Conceptual adaptation effects in the right MFG” in Discussion) is an automatic process. This automatic process resulted in a deactivated BOLD signal in the right MFG in the RP condition (Fig. 3). Thus, the mismatch between physical target-prime incongruity on the one hand and conceptual congruency on the other, elicited by target “pig” in RP, prolonged RTs (reflecting the cost of controlled processes) but had no effect on the automatic conceptual repetition suppression in the right MFG (Fig. 3).

### Possible refractoriness confounding effects

In this study we used a modified version of an original protocol controlling for refractoriness in oddball designs [61]. Although the protocol employed here does control for stimulus properties, it does not fully control for differential refractoriness. Specifically, the targets in CTR can also occur in the varying sequence not as targets (Fig. 1, blue circles). This could potentially contribute to less auditory cortex activation elicited by targets in CTR as compared to targets in RP. However, the effect of including physically identical stimuli to the targets in CTR was probably negligible. The reasoning is as follows.

The physically identical stimuli to targets which did not follow primes (Fig. 1, blue circles) appeared in CTR within a context of varying and not homogeneous repeating stimuli. On average (across all 12 versions of CTR sequences) the gap size between any two consecutive stimuli which were physically targets (red and blue circles, Fig. 1, lower panel) was ∼2.9 stimuli (with an SOA of 1 sec). Thus, the transition from a target to a short train of physically varying stimuli (i.e. fillers, isolated primes, Fig. 1) caused at least partial recovery from adaptation. This explanation holds for the N1 refractory effect [31] but is also applicable for the fMRI results. Specifically, it was previously found that even at short SOAs (2–4 msec) the refractory effect was present for congruent motion stimuli but was largely absent for incongruent stimuli [62]. By the same token, the inclusion of varying stimuli should at least elicit a partial recovery from the refractory effect reflected in the BOLD signal caused by the occurrence of two consecutive target stimuli.

### Conclusion

To the best of our knowledge this is the first study that demonstrates dissociated activity between the right MFG and MTG bilaterally as a function of the effect of semantic overlap on repetition priming. In the left MTG the introduction of the target elicited release of perceptual adaptation regardless of the identity of the target and the preceding prime. Both the right MTG and MFG were sensitive to sequence regularity extraction and its verification. However, only the right MFG was sensitive to the conceptual relations between the prime and the target that resembled categorical relations while deactivations in this region reflected an interaction between the magnitude of semantic overlap (OP/PO) and condition (RP/CTR).

This interaction in the right MFG could be explained by conceptual adaptation effects alone or by integrating conceptual adaptation effects into a competition model between sensory and semantic information (see “A hierarchical model of processing priming effects” in Discussion). Whereas previous models of intelligible speech processing underlie a trade-off between acoustic sensitivity and intelligibility, our results are compatible with a model framework of bottom-up processing which relies on differential weighting of perceptual and conceptual features when sensory information is channeled into higher-level brain regions.
